# Gain of survival signaling by down-regulation of three key miRNAs in brain of calorie-restricted mice

**DOI:** 10.18632/aging.100276

**Published:** 2011-02-27

**Authors:** Amit Khanna, Senthilkumar Muthusamy, Ruqiang Liang, Harshini Sarojini, Eugenia Wang

**Affiliations:** ^1^ Department of Biochemistry and Molecular Biology, University of Louisville School of Medicine, Louisville, KY 40202, USA; ^2^ Gheens Center on Aging, University of Louisville School of Medicine, Louisville, KY 40202, USA

**Keywords:** MicroRNA, Aging, Bcl-2, Bax, apoptosome complex, miRNA-34a, miRNA-30e, miRNA-181a-1*, cognitive robustness, apoptosis and caspase

## Abstract

The decline in cognitive robustness with aging can be attributed to complex genetic pathways involving many cellular dysfunctions, cumulative over time, precipitating in frailty and loss of wellness in the elderly brain. The size and health of the neuronal cell population determines cognitive robustness in mammals. A transgenic mouse model over-expressing Bcl-2 has been shown to rescue neurons from naturally occurring cell death (NOCD). Here we show that in the brain of calorie-restricted (CR) mice, there is an age-dependent decreased expression of microRNAs mmu-miR-181a-1*, mmu-miR-30e and mmu-miR-34a, with a corresponding gain in Bcl-2 expression, and decreases in pro-apoptosis genes such as Bax and cleavage of Caspases. Functional characterization shows that these miRNAs repress Bcl-2 expression by the 3'UTR reporter assays, accompanied by loss of this gene's endogenous expression, and a gain in pro-apoptosome-specific proteins. Over-expression of these miRNAs increases the rate of apoptosis, accompanied by a decline in Bcl-2 expression in miRNA-transfected mouse and human cell lines. We report here that down-regulation of miR-34a, -30e, and -181a permits their shared target gene expression (Bcl-2) to remain at a high level without post-transcriptional repression, accompanied by concomitant low levels of Bax expression and Caspase cleaving; this chain event may be a part of the underlying mechanism contributing to the gain in neuronal survival in long-lived CR-fed mice.

## INTRODUCTION

Most old-age perils, such as neurodegenerative, cardiovascular, and metabolic diseases, are accompanied by physiologic decline determined by the derailment of underlying molecular signaling networks at the cellular level. Cerebral aging is accompanied by a loss of synaptic contacts and neuronal apoptosis, leading to changes in cognitive function. The aging process and the related dysfunction are compounded by the combination of oxidative, metabolic and excitotoxic stress [1]. The oxidative stress may also be involved in mitochondrially mediated apoptotic pathways, accumulating calcium in aged neurons [2-5]. Age-related changes in the expression of genes involved in energy metabolism, oxidative stress, and innate immunity are counteracted by calorie restriction (CR) [6]. CR has an established neuroprotective role in the brain by up-regulating anti-apoptotic regulatory proteins that may prevent neuronal death [7]. Both CR-fed and genetically modified dwarf mouse brains manifest a significant delay in aging, and improved cognitive function [8]. As the molecular mechanisms involved may not be completely similar in CR-fed and genetically modified mice, detailing the post-transcriptional regulation of genes involved in CR-affected signaling pathways should facilitate the decoding of the divergence between these two means of extending longevity and cognitive robustness [9].

The imbalanced equilibrium between up-regulated microRNAs suppressing unwanted gene expressions and down-regulated microRNAs whose targets genes are important for cellular function has been noted with regard to the normal aging process in brain [10]. These findings suggest that the caloric restriction-derived gain of cognitive robustness may be due to the absence, reduction and/or delay of this imbalance paradigm. Here we report that this is indeed the case; in particular, our results show that three microRNAs (miRNAs), mmu-miR-181a-1*, mmu-miR-30e and mmu-miR-34a, show no age-dependent up-regulation in CR animals, seen in *ad lib-*fed controls, nor reciprocal up-regulation of their target, the Bcl-2 gene. The down-regulation of these three miRNAs was initially identified by array screening, and validated by RT-PCR and *in situ* hybridization profiling in brains of older, CR-fed mice, compared to their *ad lib*-fed littermates. Parallel protein profiling shows an age-dependent increase of Bcl-2 in brains of older calorie-restricted mice. Functionally, all three microRNAs are capable of repressing the reporter expression of Bcl-2 3'UTR construct; the transfected cells are shown to lose this pro-survival gene and gain pro-apoptosis gene expression, with increased death rates in both mouse and human cell culture models.

In summary, our data suggest that caloric restriction may induce the loss of age- dependent increase of key miRNAs, and this loss may in turn permit Bcl-2 to remain elevated in the brains of older, CR-fed animals, resulting in the loss of pro-apoptosis signaling and gain of neuronal survival. Thus, loss of deleterious up-regulation of these miRNAs may be the underlying mechanism contributing in part to the gain of robustness in CR-fed mice.

### MiRNA expression targeting Bcl-2 in calorie-restricted mouse brain

**Figure 1.**
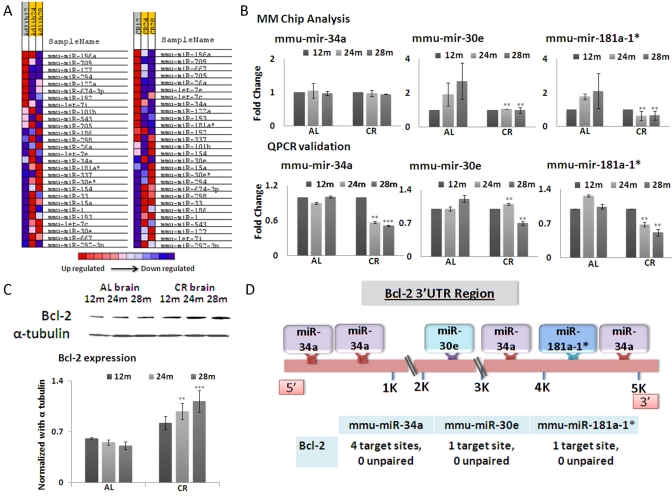
(**A**) Based on microRNA microarray chip data, a heat map indicating the expression levels of miRNAs with respect to age and mouse diet category (*Ad libitum*, AL and Calorie restricted, CR) in brain tissues was generated. Intensity of color represents relative expression, with blue representing decreased expression, and red representing increased expression. The comparison is drawn on the basis of expression among the mouse categories. (**B**) Graphical representation of miRNA (mmu-miR-34a, mmu-miR-30e and mmu-miR-181a-1*) expression in brain tissues, which exhibit the greatest fold changes among 12, 24 and 28 months in CR-fed and *Ad lib* mice. Samples are represented by age in months (12, 24, or 28). AL and CR for *Ad lib* and calorie restricted respectively. (**C**) Western blot analysis of Bcl-2, with alpha-tubulin as normalization control. The histogram shows average densitometry measurements of Bcl-2 after normalization. (**D**) Schematic diagram showing miRNA binding sites on the 3'UTR region of the Bcl-2 gene. (**p<0.01, **p<0.0001 in panels **B** and **C**; all histograms represent average ± std.dev.)

The comparative expression of the miRNAs of interest was screened by miRNA microarrays (MMChips) in brain samples of CR- and ad lib-fed mice. The brain tissues were obtained from the National Institute on Aging tissue bank; C57/black 6 mice were calorie- restricted to 60% of normal ad lib caloric intake, according to the standardized regimen, starting at 4 months of age. By 12 months of age, the CR impact is fully established; therefore this age group was selected along with two older groups, 24 and 28 months (Supplementary Figure 1A). For each group, total RNA from brain tissues of three mice was used, to control for inter-animal variations. In addition, three MMChips were used for each mouse brain RNA sample, to generate statistically significant results of 9 datasets per age group. The Significance Analysis of Microarray(SAM) software listed miRNAs showing significant change (Figure 1A). MicroRNAs exhibit variations in expression in CR-fed mouse brain tissues, compared to their ad lib-fed counterparts, with inverse expression of age-dependent patterns (Figure 1A).

Among these, three microRNAs, mmu-mir-181a-1*, mmu-mir-34a and mmu-mir-30e, exhibit the most significant down-regulation in brains of CR-fed mice, in an age-dependent manner, compared to *ad lib*-fed mice (Figure 1B). The expression profiles of these three microRNAs were further confirmed by quantitative real-time PCR (qRT-PCR) (Figure 1B). These three miRNAs were selected because they manifest correlated fold changes at 24 and 28 months between CR- and *ad lib*-fed mice. All three microRNAs display significantly decreased expression in CR-fed mice at 28 months of age, compared to *ad lib*-fed mice. A disparity exists between MMChip results and qPCR in miR-30e expression, when 24 month old CR-fed mice are compared to 24 month old *ad lib*-fed control mice; the array results show decreased expression, whereas qPCR results show increased expression in 24-month CR-fed mice (Figure 1B). The qPCR data also show a significant difference in miR-34a expression between CR- and *ad lib*-fed mice at 28 months of age, whereas array data point to almost similar expression levels at that age. These anomalies seem to be based on the fact that microarray screening is less sensitive, and the analyses are based on signal strength from hundreds of loci on the arrays. Even after normalization with negative and positive controls, the assay is less sensitive than qPCR assay, which quantifies a single miRNA at a given time. Interestingly, a common denominator among all these three miRNAs is the age-dependent decrease in expression in CR-fed compared to *ad lib*-fed mice.

The miRBase targets version 5 database (http://microrna.sanger.ac.uk/) reported a set of nine genes which are targeted by all three miRNAs (Figure 1B); three other databases were employed to further confirm the predicted relationship between these miRNAs and these target genes. Bcl-2 was a consistent result from all the databases (Target Scan, Pictar and RNA 22). This analysis pointed to the fact that even human Bcl-2 may be a target for these miRNAs, as the seed sequences of these three miRNAs are well conserved between mice and humans (data not shown). In *ad lib*-fed mouse brain tissues Bcl-2 expression levels show an age- dependent decline (Figure 1C). In CR brain tissues, Bcl-2 expression is not only maintained, but rather increases as the mice reach the advance age of 28 months. This persistent increase in expression levels with age is inversely related to the reduced expression of miRNAs at the age of 28 months (Figure 1C).

We further analyzed the number of target sites for these miRNAs on the 3' UTR regions of the predicted target genes. Based on these exercises of algorithmic prediction, the 3'UTR of Bcl-2 and binding sites of the three miRNAs are depicted in the schematic diagram (Figure 1D).

### In situ detection of miRNAs and Bcl-2 in calorie-restricted mouse brain tissues

**Figure 2.**
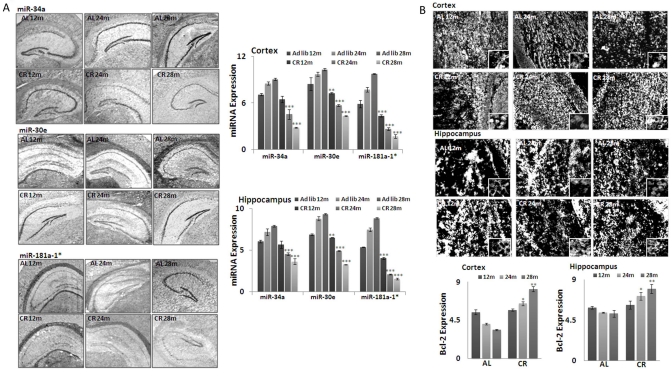
Expression of three key MicroRNAs and Bcl-2 in CR mouse brain tissues (**A**) *In situ* hybridization (ISH) detection of miRNAs (mmu-miR-34a, mmu-miR-30e and mmu-miR-181a-1*)in mouse brain tissues from both CR- and *Ad lib-*fed mice. Note increased hybridization signal of these miRNAs in the *Ad lib* group across all age groups, compared to CR. Graphical representations of densitometric analysis of miRNA (mmu-miR-34a, mmu-miR-30e and mmu-miR-181a-1*) hybridization in cortex and hippocampal regions. (**B**) Increased immunohistochemical staining of Bcl-2 in cortex and hippocampal region of CR, compared to *Ad lib* mice brain. Graphical representations of densitometric analysis of miRNA (mmu-miR-34a, mmu-miR-30e and mmu-miR-181a-1*) staining in cortex and hippocampal regions. (**p<0.01, **p<0.0001; all histograms represent average ± std.dev.)

MicroRNA -181a-1*,-34a and -30e expression patterns at different ages in both CR- and *ad lib*-fed mice were determined by *in situ* hybridization (ISH), using LNA (locked nucleic acid) probes (Figure 2A), in coronal serial sections of 10 μm thickness from 12, 24 and 28 month old brains. The densitometry values from the acquired images were normalized with the values from a hybridization control where a scrambled LNA probe was used. The expression profiles were consistent with our array screening and qPCR validation results. All three miRNAs showed reduced staining intensity in CR-fed mouse brains, compared to *ad lib*-fed mice; this difference was seen not only in cortical, but also in hippocampal, regions (Figure 2A).

The predicted target for these miRNAs, Bcl-2 expression, was detected by immunohistochemistry, as described in Methods. Increased staining of Bcl-2 was observed in CR-fed, compared to *ad lib*-fed, mice, in an age-dependent manner (Figure 2B). Densitometry values from the acquired images were normalized with values from a blank control (picture not shown). Cortical and hippocampal regions evenly show the elevated expression of Bcl-2 in CR- fed mice, in an age-dependent manner. Taken together, the correlated inverse expression of these miRNAs and Bcl-2 was validated by *in situ* hybridization and immunohistochemistry study.

### MiRNA-dependent down-regulation of Bcl-2 in human and mouse cells

**Figure 3.**
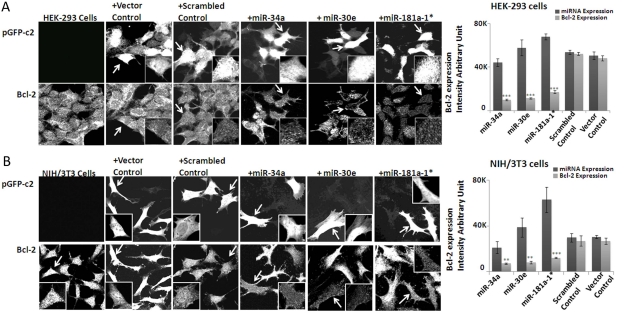
Effect of over-expression of microRNAs on Bcl-2 expression in HEK-293 and NIH/3T3 cells Immunocytochemistry analysis of miRNA (mmu-miR-34a, mmu-miR-30e and mmu-miR-181a-1*) suppression of endogenous Bcl-2 in both 293 (**A**) and NIH-3T3 (**B**) cell strains. The cells transfected with plasmid showing fluorescence and Bcl-2 immunostaining are from the respective identical fields. In each set (HEK-293 and NIH-3T3 cells), miRNA-transfected cells have significant Bcl-2 staining reduction, compared to Bcl-2 signal in neighboring cells lacking miRNA transfection. This pattern does not pertain in scrambled plasmid-transfected 293 or NIH-3T3 cells; scrambled plasmid-transfected cells show Bcl-2 staining intensity identical to un-transfected cells. The histograms show the quantification of fluorescence for plasmid transfection and the corresponding Bcl-2 expression in the cells. (**p<0.01, **p<0.0001; all histograms represent average ± std.dev.)

To evaluate the repression of Bcl2 by any of the three microRNAs, we used transfection to over-express miR-34a, -30e, or -181a in human embryonic kidney cells (HEK-293 cells) and mouse fibroblast cells (NIH/3T3 cells). The fact that miRNA binding sites on the 3'UTR region of Bcl-2 are conserved between mice and men gave us the opportunity to test this relationship in a mouse-independent system. Our results show that indeed Bcl-2 expression is repressed in all three miRNA-transfected cultures, by individual cell immnocytochemistry of transfected cultures with anti-body to this protein (Figure 3A-B). The densitometry values from the acquired images provide quantitative indices for this repression effect, plotted as histograms of miRNA expression in relationship to the repressed level of Bcl2 in these transfected cells.

### MiRNAs effect Bcl-2/Bax ratio favoring pro-survival signaling

**Figure 4.**
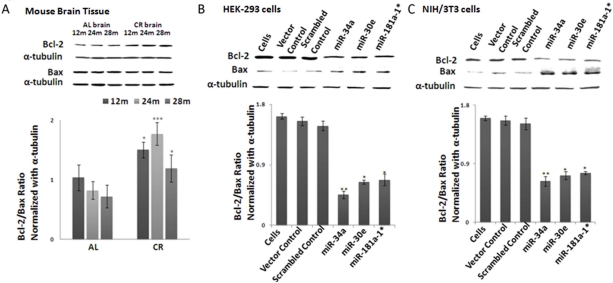
Bcl-2/Bax ratios in calorie-restricted mouse brain tissue and over-expressed miRNA- transfected cell lines Western blot analysis of Bcl-2, Bax and alpha-tubulin as normalization control. (**A**) In CR-fed mouse brain tissue, increased Bcl-2 is observed compared to *Ad Lib* (AL)-fed mice, while Bax expression stays constant. Increased Bcl-2/Bax ratio is observed in CR-fed mice, compared to *Ad Lib*-fed mice. Histogram represents ratios of Bcl-2 and Bax expression. (**B**) In miRNA- (mmu-miR-34a, mmu-miR-30e and mmu-miR-181a-1*) transfected HEK-293 cells, Bcl-2/Bax ratio declines, as Bcl-2 expression is considerably diminished in miRNA-transfected cells, compared to scrambled and vector controls. Histogram represents ratios of Bcl-2 and Bax expression. (**C**) In line with results in HEK-293 transfected cells, miRNA-transfected NIH/3T3 cells also exhibit a decline in Bcl-2 expression, resulting in a decline of the Bcl-2/Bax ratio. Histogram represents ratios of Bcl-2 and Bax expression. (**p<0.01, **p<0.0001; all histograms represent average ± std.dev.)

Since Bcl-2 is an anti-apoptotic, and Bax a pro-apoptotic, gene, their ratio is a profound indicator of cell survival; this survival ratio can be altered either by increased Bcl-2 expression or decreased Bax expression. In CR-fed mice the Bcl-2/Bax ratio showed an increased trend in an age-dependent manner, compared to *ad lib*-fed mice (Figure 4A). To validate this antagonistic pattern between Bcl2 and Bax protein levels, we examined their ratios in microRNA-transfected HEK-293 and NIH/3T3 cultures. Total cellular protein fractions were isolated, as described in Methods. Decreased expression of Bcl-2 in miRNA-transfected cultures indeed led to a decline in Bcl-2/Bax ratio (Figure 4 B-C); densitometry values from the acquired Western blot images were normalized with values from alpha-tubulin blots as the loading control. Taken together, each of these three miRNAs is able to repress Bcl-2 in transfected cells, with subsequent increase of Bax proteins, thus resulting in a reduced Bcl2/Bax ratio. Finally, these results provide validation that Bcl-2 is a shared target for silencing by all three miRNAs at the post-transcriptional level.

### Altered expression of apoptosome-specific proteins Caspase-9 and Caspase-3

**Figure 5.**
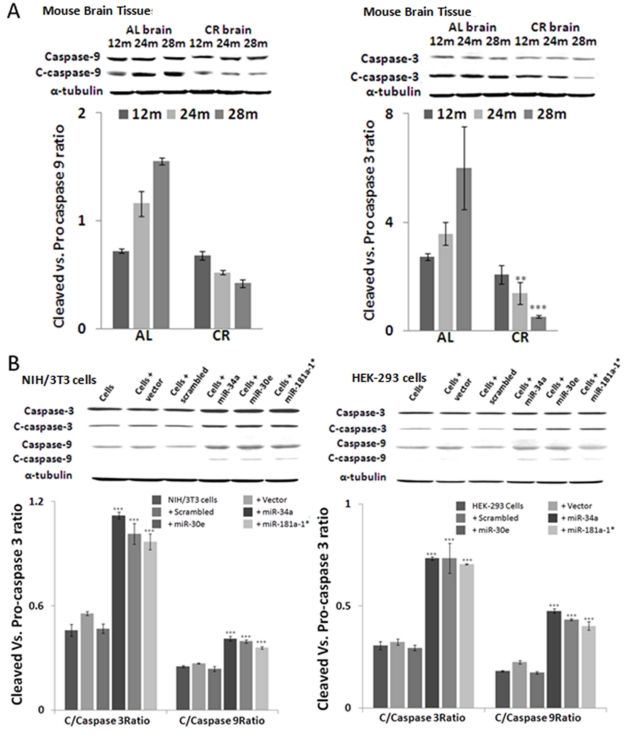
Altered expression of active apoptosome-specific proteins in calorie-restricted mouse brain tissue and in over-expressed miRNA cell lines Cleaved Caspase-9 (C-Caspase-9) and cleaved Caspase-3 (C-Caspase-3) are important components of the apoptosome complex. (**A**) In calorie-restricted (CR)-fed mouse brain tissues, levels of c-Caspase-9 and -3 decline in an age-dependent manner, compared to *Ad Lib* (AL)-fed mice. (**B**) Over-expression of three miRNAs in HEK-293 and NIH/3t3 cells produces a significant increase in cleaved form as compared to scrambled and vector alone controls. Increase in these apoptosome-specific proteins indicates the pro-apoptotic effect of these miRNAs. (**p<0.01, **p<0.0001; all histograms represent average ± std.dev.)

To validate the effect of Bcl-2 on apoptosome-mediated apoptosis, we examined the expression of Caspase-9 and Caspase-3. In line with Bcl-2 expression, the expression of cleaved Caspase-9 and Caspase-3 is significantly reduced in age-dependent manner from young to old age groups in CR-fed, compared to ad lib-fed, mouse brain samples (Figure 5A). Western blot assays were done using brain tissue samples from three different animals from CR- and ad lib- fed mice from each age group. For Western blot, in situ and qPCR assays, the specimens used were from the same mice, to maintain homogeneity and avoid inter-animal variation. The level of α-tubulin expression was used as the internal control for normalization of values of these two proteins.

### MiRNA-mediated repression of Bcl-2 3'UTR and elevated apoptosis in cell lines

To address the cascade effect from over-expression of the three microRNAs to repress Bcl-2 expression, with subsequent increase of Bax and downstream corresponding increase in Caspase activities, the miRNA-transfected HEK-293 and NIH/3T3 cultures were further examined by Western blot analysis with antibodies to Caspases, whose autocatalytic cleavage is essential to execute apoptotic death. Our results show that in transfected cells, the decreased Bcl-2/Bax ratio is accompanied by a significant increase in cleaved Caspase-9 and Caspase-3 (Figure 5B). Further validation of the three microRNAs’ ability to repress Bcl-2, and induce the chain reaction of an increase in Bax and Caspase cleavage, was achieved by direct repression assays with co-transfection experiments, where miR-34a, -30e, or 181a-1* expression plasmids were used to co-transfect along with cloned 3'-UTR reporter construct in HEK-293 cells. At 72 hours, the green fluorescence corresponding to the miRNA constructs can be observed, with fading red fluorescence of the reporter expression of 3'UTR constructs, compared to co-transfection experiments using control vector carrying a scrambled sequence. Image analysis of the intensities between the miR-plasmid and reporter constructs was performed; repression of the Bcl-2 3'UTR reporter intensities was significant in the miR-plasmid co-transfected cells, but not in their control counterparts.

Further functional impact of these miRNAs’ repression of bcl2 expression was performed by examining putative increases of apoptotic death in the transfected cells. MiRNA- transfected HEK-293 and NIH/3T3 cells were subjected to apoptosis inducers such as staurosporine, a general protein kinase inhibitor isolated from the bacterium *Streptomyces staurosporeus.* Our results show that indeed transfected cells show dosage-dependent decrease in LD_50_. Since the transfection assays rarely acquire 100% efficiency, cell death percentages were calculated based on the positively transfected cell number, rather than the total cells in the cultures; the increase of apoptosis in cells with transduced microRNA expression indeed corresponds to the decrease of LD_50_ indices.

Taken together, our work has shown that: during aging, miR-34a, -30e, and -181* are not up-regulated in CR brain, while pro-survival gene Bcl-2 is maintained at the same level in older mice as their younger counterparts, accompanied by a decrease of pro-apoptosome proteins such as Bax and Caspase 9 cleaved products. Functional validation of the three microRNAs’ silencing effect on Bcl-2, with subsequent induction of pro-cell death signaling, was documented by transfection studies, and suggests the role of these miRNAs in apoptosis. Thus, during aging, down-regulation of the three microRNAs may indeed reflect a gain of survival signaling in neurons of older CR mice.

## DISCUSSION

In the present study, our data report that lead microRNAs (mmu-miR-181a-1*, mmu-miR-30e and mmu-miR-34a) are not up-regulated in the older CR mouse brain, compared with their *ad lib* counterparts. This corresponds to Bcl-2 expression maintaining at a high level, and down-regulated proteins of apoptosome formation. Functional assays show that indeed the three miRNAs can repress Bcl-2 and increase pro-apoptosis gene expression and cell death. The CR regimen seems to ablate the ill effects of aging by down-regulating these miRNAs in an age- dependent manner. Most interestingly, our results demonstrate that not only one, but three microRNAs targeting the pro-survival gene, Bcl-2, are all down-regulated in older CR brains. This suggests that the caloric restriction regimen may affect the group dynamics of the three microRNAs in unison, and the ablation of their up-regulation could be the pivotal post-transcriptional regulation to reduce the trend of increased cell death in brain during aging. Down-regulated miR-34a, -30e, and -181a-1*, together then not only provide neuronal biomarkers for the CR brain, but also their impact on the shared target, Bcl-2, may be the cornerstone to unravel the complex pathways involved in neuronal survival signaling.

**Figure 6.**
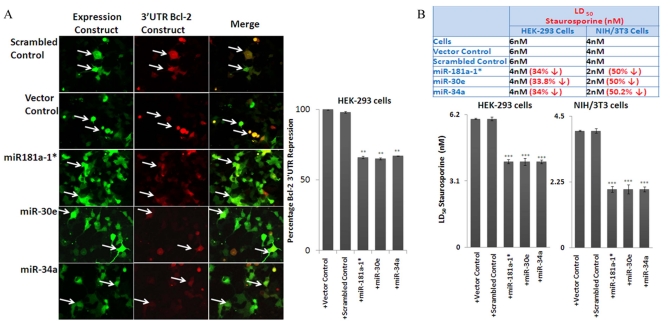
Effect of miRNAs on Bcl-2 3'UTR and rate of apoptosis in cell lines, using transfection assays (**A**) miR-34a/−30e/181a-1* and Bcl-2 (3'UTR) co-transfected 293 cells express reduced red fluorescence, which shows that the 3’ UTR of Bcl-2 is repressed by the input miRNAs, but not by scrambled control and Bcl-2 (3'UTR) co-transfected cells. This shows that these miRNAs indeed suppress the target protein (red fluorescence protein) through the 3'-UTR of Bcl-2. This effect is absent when a plasmid carrying a scrambled sequence is used (indicated with arrows). (**B**) In line with the increase of apoptosome-specific proteins in the over-expression assay for these three miRNAs, over-expression in HEK-293 and NIH/3T3 cells contributes to a decline of the LD_50_ dose of Staurosporine, thus more cell death at lesser concentration; this effect on the LD_50_ dose is not present in scrambled and vector alone controls. (**p<0.01, **p<0.0001; all histograms represent average ± std.dev.)

**Figure 7.**
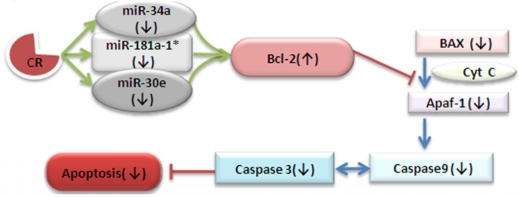
Calorie restricted mediated alteration of survival signaling This schematic diagram shows the contribution of three key down-regulated miRNAs in CR mice for up-regulation of Bcl-2, effecting the process of cell survival signaling through Caspases-9 and -3, resulting in a decline of apoptosis.

Apoptosis suits itself into antagonistic pleiotropy theory and as it has deleterious effects in aging neurons while it is needed during development where it removes neurons that don't integrate into the growing neuronal network [11]. However, after the development of the neuronal network into the mature nervous system, the fight of neurons for survival and healthy existence with plasticity becomes anti-apoptotic [12]. In the mitochondrial pathway of apoptosis, intracellular signals result in releasing cytochrome *c* from mito-chondria, which binds to the adaptor protein APAF-1 (apoptotic protease-activating factor-1), which further leads to the activation of Caspase 9 and subsequently Caspase 3; these are the proteases responsible for disintegration of the cell, Figure 7 and [13, 14]. Our results suggest a post-transcriptional paradigm by CR regimen for down-regulation of three vital microRNAs, miR-34a, -30e, and -181a, with consequential loss of Bcl-2 repression and downstream Bax/Caspase decrease, a scenario favoring neuronal survival. The cascade effect of the decrease of the three microRNA expressions in the CR-treated mouse brain is Bcl-2 not showing the usual age-dependent decrease, with decreased Bax expression and Caspase activity. This microRNA-directed impact on survival signaling is due to the fact that Bcl-2 as a pro-survival gene is a member of the anti-apoptotic group, which binds to and inhibits the action of multi-domain pro-apoptotic proteins like Bax [15]. Moreover, the up-regulation of Bcl-2 expression leads to down-regulation of Bax, and interferes with the release of cytochrome c from the mitochondrial intermembrane space into the cytosol, where it associates with Caspase 9 and Apaf1 to form the apoptosome complex, thus effecting apoptosis [16, 17]. Figure 6 C elucidates the role of elevated Bcl-2 expression because of the decline in these three miRNAs’ expression in CR-fed mouse brain tissues. This increase in Bcl-2 expression leads to altered survival signaling, leading to disrupted apoptosome complexes.

It is well known that age-associated neurodegeneration is associated with increasec neuronal apoptosis, as one mechanism contributing to cognitive dysfunctions [18-21]. Caspase 3 and Bax up-regulation has been observed in patient samples and in classic animal neurodegenerative models such as stroke, AD (Alzheimer's disease), HD (Huntington's disease) and PD (Parkinson's disease) [22, 23]. The Bcl-2 protein resides in the outer mitochondrial wall, and acts as an anti-apoptotic factor by controlling mitochondrial permeability, thus regulating apoptosis [24]. More evidence of the interplay of Bcl-2 and Bax in these diseases comes from the increased neuronal survival in mouse models that either have Bcl-2 over-expression or Bax knockout [25-30]. Bcl-2 is known to protect neurons against Ca2+-mediated death [31]. Previous evidence of miRNA impact on cell survival mechanisms has been shown with miR-15 and miR-16 targeting Bcl-2 mRNA and inducing apoptosis [32, 33]. Therefore the down-regulation of miRNAs that target pro-survival genes may be interpreted as potential therapeutic candidates for preventing apoptotic death in neuronal cells, thus contributing to cognitive robustness. Little is known as to how post-transcriptional regulation of cellular signaling underlies the mechanism of the CR impact on extended life span. Our present work is an early example of how non-coding RNAs such as microRNA can modulate key gene expression, specifically the absence of the normal up-regulation of key microRNA expression. This absence indirectly results in the presence of genes whose function is essential to maintain the wellness of neurons. The example of the down-regulation of three lead microRNAs, with the corresponding maintenance of pro-survival genes and the cascade effect leading to the lack of apoptosis-inducing factors, opens the door to a fertile ground of discovery of CR impact on post-transcriptional modification.

Mechanistically, expression of non-coding RNAs such as microRNA follows molecular regulation like that of coding genes, involving binding of a transcriptional factor (TF) to a promoter region for the specific miRNA to be transcribed, with feed-forward and feed-back loop modulation, *etc.* Intervention by caloric restriction probably induces putative crucial TF/miR activation, with complex biochemical regulation [10]. Obviously, identification of TFs responsible for the activation of these three microRNAs, miR-34a, -30e, and -181a, will be an essential first step in our quest for the impact of CR on their down-regulation. Success of this approach will open the possibility that CR mimetics could be developed, so as to endow wild-type ad lib-fed animals with the same benefits of not having these microRNAs up-regulated. Future work in this direction will provides leads such that intervention in the later stagesof life may be approached by post-transcriptional modification of unwanted microRNA up-regulation. Thus, pro-survival Bcl-2 expression would be maintained at its optimal level, reducing neuronal cell death, the cellular contribution to cognitive robustness.

## METHODS

For comparative proteomic and miRNA expression analysis, 18 mice were divided equally into CR and AL populations across 3 age groups: 12, 24 and 28 months. All experiments were carried out in triplicate.

### MicroRNA profiling

Small RNA samples (1 μg) were labeled with digoxigenin (DIG) using the DIG Oligonucleotide Tailing Kit, 2nd Generation (Roche Diagnostics, U.S.A.) as described earlier [34]. Our own mouse miRNA DNA nitrocellulose membrane microchips (MMChips) were printed bearing 367 anti-sense mature miRNA sequences from miRBAse (http://microrna.sanger.ac.uk/Version 8.2) as previously described [35]. The membranes were scanned by an Expression 1680 scanner (Epson, U.S.A.), and data was acquired using Array-Pro Analyzer 4.5 software (Media Cybernetics, MD, U.S.A). Net intensity was derived from whole cell area measurement, and corrected using mean intensity of ring background of surrounding spots. Microarray data analyses were performed with SAM software, version 3.02 (Significance Analysis of Microarrays, Stanford University, CA), including pairwise comparison (T statistics) between age groups. Kolmogorov-Smirnov statistics were generated by Gene Set Enrichment Analysis (GSEA) software [36].

### Mouse tissue samples

Tissue samples were obtained from the National Institute of Aging (http://www.nia.nih.gov/ResearchInformation/ScientificResources/AgedRodentTissueBankHandbook/RodentTissueBankResourcesInfo.htm) from CR- and AL-fed mice, at age groups of 4, 12, 24, and 28 months. The tissue samples were rapidly frozen at −80°C for analysis. Tissue samples were processed for protein, RNA extraction and *in situ* experiments as described below. Animal tissue related processing and experiments were done in accordance to the approved institutional (University of Louisville) biosafety board protocol #05-001.

### Total and small RNA extractions

For total RNA extraction, the frozen tissue blocks were homogenized with Trizol according to the manufacturer's instructions. Small RNA enrichment was done using incubation with PEG 8000, as described previously [37].

### Protein extraction

The frozen tissues were diced, then homogenized in two volumes (g mL–1) of RIPA buffer (150 mm NaCl, 10 mm Tris, pH 7.2, 0.1% SDS, 1.0% Triton X-100, 1% deoxycholate, 5 mm EDTA, pH 8.0) containing 1× protease inhibitor (Calbiochem, San Diego, CA) and centrifuged at 10,000 × g for 10 min. The supernatant containing the proteins was harvested. The Bradford method was used to quantify total protein concentration with BioRad reagents (BioRad, Hercules, CA).

### QRT-PCR validation

Quantitative real-time PCR was carried out using Taqman miRNA assay reagents to verify miRNA expression profiles. Two hundred nanograms of total RNA were reverse transcribed to cDNA; the real-time PCR reactions were performed on a 7500 Fast System Real-Time PCR cycler (Applied Biosystems, Foster City, CA), according to the manufacturer's instructions. MicroRNA expression fold changes between ages across the groups were calculated using the delta Ct method, using RNU44 for normalization.

### Western Blotting

The brain tissues (12, 24, 28 month *ad lib*- and CR-fed mice, n=3 each) were homogenized as described previously [34]. Following this procedure, membranes were incubated overnight at 4°C with primary antibodies: rabbit anti-Bcl-2, rabbit anti-Bax, rabbit anti-Caspase-3 and rabbit anti-Caspase-8 antibodies (sc-783, sc-6236, sc-7148 and sc-7890; Santa Cruz Biotechnology Inc, Santa Cruz, CA) and rabbit anti-Caspase-9 antibody (ab32539; Abcam Inc., Cambridge, MA). Blots were developed as described before [34].

### Tissue processing for *in situ* hybridization and immunohistochemistry

The frozen brain tissue blocks were prepared by placing them in a cutting mold on dry ice, covered with Optimal Cutting Temperature (O.C.T.) compound (Sakura, Torrance, CA). The blocks were kept at −80 °C overnight; then 10 μm sections were collected in the coronal plane by cryostat (Leica, Solms, Germany), and mounted onto Superfrost^®^Plus microscopic slides (Fisher Scientific, Pittsburgh, PA). Slides were stored at −80°C until further use.

### MicroRNAs locked nucleic acid (LNA) *in situ* hybridization

The frozen sections were fixed with 4 % paraformaldehyde (PFA) for 30 min at room temperature, washed in 0.1 M phosphate-buffered saline (PBS) for 20 min, and treated with acetylation solution (92.8 % diethylpyrocarbonate (DEPC)-treated water, 1.3 % triethanolamine, 0.2 % HCl, 5.7 % acetic acid) for 20 min. The *in situ* hybridization was performed for miR-34a, miR-30e and 181a-1* as described previously [34].

### Immunohistochemistry

The sections were then blocked with 10 % goat serum for 30 minutes (Invitrogen, Carlsbad, CA), and incubated with rabbit anti-Bcl-2 antibody (sc-783; Santa Cruz Biotechnology Inc, Santa Cruz, CA) overnight at 4°C. After incubation, the sections were washed thrice with PBS for 5 minutes, and incubated with Alexa fluor 555 goat anti-rabbit IgG (#4413; Cell Signaling Technology, Inc., Danvers, MA). For signal detection and analysis the procedure was followed as described earlier [34].

### Construction of Bcl-2 3'-UTR Reporter Construct and miRNA expression constructs

Mouse Bcl-2 full length 3'-UTR was cloned into ptdTomato-C1 vector from Clontech (Mountain View, CA), following the gap repair method [38]. Briefly, upstream and downstream miniarms were amplified from mouse C57BL/6J genomic DNA using the following primers. The retrieve construct was made by cloning these two miniarms into EcoRI plus BamHI linearized ptdTomato-C1, following the Circular Polymerase Extension Cloning method [39]. After sequencing verification, the retrieve construct was linearized with EcoRV and electroporated into recombineering-competent bacterial artificial chromosome (BAC) clone containing the Bcl-2 3'-UTR genomic sequence. The BAC clone was made recombineering-competent by transformation with pKD46 [40]. Bcl-2 3'-UTR in ptdTomato-C1 was selected by kanamycin resistance, and verified by full coverage sequencing.

Expression constructs of mouse miR-34a, -30e, -181a, and miR-93 over were made by cloning miRNA precursor sequences with a flanking genomic sequence amplified from mouse genomic DNA (C57BL/6J) into pCDH-CMV-MCS-EF1-copGFP vector (System Biosciences, Mountain View, CA). The primers used for miRNA-specific primer sequences were mmu-miR-34a-Fwd: TGCTGTACCCTGCTGCTT, mmu-miR-34a-Rev: ACTCCAGACCCAGTGCCTC, mmu-miR-93-Fwd: TTGAATCCATGAGGGCTAGG, mmu-miR-93-Rev: TGTTCAGCTGTCCTGTGAGG, mmu-miR-30e-Fwd: GCAAGCCCTGGGTCACCTCC, mmu-miR-30e-Rev: AGCAATGCCTGACTGCATCACA, mmu-miR-181a-Fwd: GCCAAGCTCTGGGCTGGAGG and mmu-miR-181a-Rev: TCGGGTCCTGCTTTGCGG TT. The miRNA expression constructs were grown in Stbl3 strain (Invitrogen, Carlsbad, CA).

### Cell transfection/co-transfection studies

Human embryonic kidney (HEK) 293 cells and NIH/3T3 cells (ATCC, Manassas, VA) were used for transfection/co-transfection experiments with the cell line nucleofector kit V by Nucleofector System (Lonza Walkersville Inc., Walkersville, MD), according to the manufacturer's instructions. In brief, 1 × 10^6^ cells were suspended in 100 μL nucleofector solution, and transfected with 5 μg of plasmids (vector control, scrambled control, mmu-miR-34a, mmu-miR-30e or mmu-miR-181a-1*). For co-transfection, the Bcl-2 3'UTR construct was co-transfected with vector alone, scrambled control, miR-34a, miR-30e and miR-181a-1* (1:1 ratio). The procedure followed has been described previously [34].

### Apoptosis Assay

Human embryonic kidney (HEK) 293 cells and NIH/3T3 cells (ATCC, Manassas, VA) were used for transfection/co-transfection experiments with the cell line nucleofector kit V from Nucleofector System (Lonza Walkersville Inc. Walkersville, MD) according to the manufacturer's instructions. MiRNA expression plasmids were transfected, and transfection efficiency was calculated. Various doses of Staurosporine (2nM to 10nM) were used, and LD_50_ was calculated based on the cell death ratios in miRNA-transfected and their respective control transfected cells. LD_50_doses for miRNA transfected cells were calculated to determine the effect of miRNA on cell survival. Cells were stained with Tryphan blue as per the manufacturer's instructions http://cellgro.com/.

### Statistical analysis

All tests were performed using SPSS Professional Edition software (IBM). Descriptive statistics including mean and s.e.m. along with one-way ANOVAs followed by multiple comparison tests and two tailed T test were used to determine significant differences. P < 0.05 was considered significant.

## SUPPLEMENTARY FIGURE

Supplementary Figure 1. Minimal change in miRNA expression between 4 and 12 months, and shared targets of miRNAs (**A**) Fold changes between CR-fed mouse brain tissues at 12 months and littermates at 4 months are identical. As the dietary regimen was started at 4 months, insignificant change in these three miRNA expressions signifies that the effect of CR becomes evident later in life. (**B**) All three miRNAs share nine targets, based on bioinformatics (RNA22 program), signifying the ‘pack-hunting’ role in which multiple miRNAs target single genes to manipulate crucial cellular mechanisms.
