# Supplementary figures and images for: Gain of survival signaling by down-regulation of three key miRNAs in brain of calorie-restricted mice
Source: Aging (Albany NY). 2011 Feb 27;3(3):223–36. doi: 10.18632/aging.100276 (PMC3091518; doi:10.18632/aging.100276)

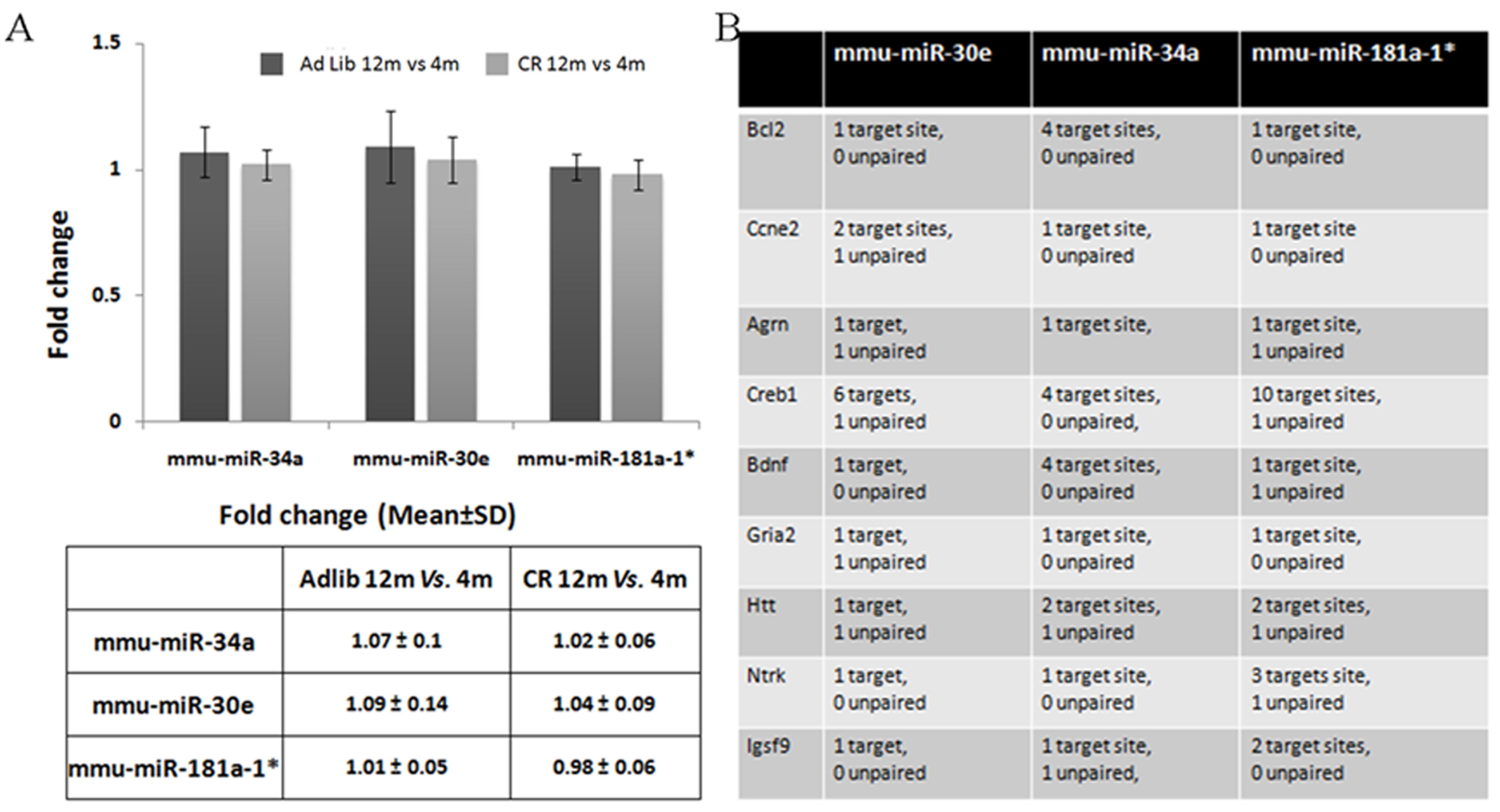

Supplement: Supplementary Figure 1. — (A) Fold changes between CR-fed mouse brain tissues at 12 months and littermates at 4 months are identical. As the dietary regimen was started at 4 months, insignificant change in these three miRNA expressions signifies that the effect of CR becomes evident later in life. (B) All three miRNAs share nine targets, based on bioinformatics (RNA22 program), signifying the ‘pack-hunting’ role in which multiple miRNAs target single genes to manipulate crucial cellular mechanisms. [file aging-03-223-s001.tif]
